# Mechanisms and Translational Potential of Plant-Derived Extracellular Vesicles in Cardiovascular Disease

**DOI:** 10.3390/cells15151416

**Published:** 2026-08-05

**Authors:** Songyan Tie, Huifang Kuang, Hang Xu, Qian Guo, Jie Li, Lingli Chen

**Affiliations:** 1 School of Integrative Traditional Chinese and Western Medicine, Hunan University of Chinese Medicine, Changsha 410208, China; 20252073@stu.hnucm.edu.cn (S.T.); 20242119@stu.hnucm.edu.cn (H.K.); 2 Hunan Provincial Key Laboratory of Vascular Biology and Translational Medicine, Hunan University of Chinese Medicine, Changsha 410208, China; 3 Medical School, Hunan University of Chinese Medicine, Changsha 410208, China; 20243832@stu.hnucm.edu.cn (H.X.); 19811802482@163.com (Q.G.); 4 Library, Hunan University of Chinese Medicine, Changsha 410208, China; 003290@hnucm.edu.cn

**Keywords:** plant-derived extracellular vesicles, cardiovascular diseases, mechanisms, targeted drug delivery, clinical translation

## Abstract

**Highlights:**

**What are the main findings?**

- PDEVs modulate multiple pathological processes in cardiovascular disease, including inflammation, oxidative stress, lipid dysregulation, endothelial injury, and cell death.
- Selected engineered PDEV systems further improve cargo delivery, lesion targeting, and local retention in experimental models.

**What are the implications of the main findings?**

- Preclinical studies suggest that PDEVs may serve as multifunctional candidates for cardiovascular therapy and drug delivery.
- Defining cargo–function relationships and improving standardization will be critical before clinical translation can be considered.

**Abstract:**

Cardiovascular diseases remain a major global health burden. Plant-derived extracellular vesicles (PDEVs) are increasingly being investigated as potential therapeutic and drug-delivery platforms for cardiovascular disease. PDEVs are natural nanovesicles carrying bioactive lipids, proteins, nucleic acids, and phytochemicals. Preclinical studies suggest that selected PDEV preparations may exert protective effects in cardiovascular disease-related models by modulating inflammation, oxidative stress, lipid metabolism, and endothelial repair. In experimental models, selected PDEVs have shown preliminary improvements in cargo stability, lesion accumulation, controlled release, and local retention through drug loading, surface ligand modification, responsive design, and integration with biomaterials. This review summarises the biogenesis, isolation, characterisation, and cardiovascular actions of PDEVs, with emphasis on their engineering and targeted delivery applications in atherosclerosis, myocardial infarction, ischaemia–reperfusion injury, vascular calcification, restenosis, and cardiotoxicity. Current challenges, including insufficient standardization, uncertain regulatory classification, unclear mechanisms, and limited pharmacokinetic and long-term safety data, are also discussed. Addressing these issues is essential for reliably evaluating the clinical translation potential of PDEVs.

## 1. Introduction

Cardiovascular diseases (CVDs) remain a leading cause of death worldwide, and their global burden continues to increase [1]. The development and progression of CVDs involve multiple interrelated pathological processes, including inflammation, oxidative stress, lipid dysregulation, endothelial dysfunction, and cardiomyocyte death [2]. Although current therapies, such as β-blockers, statins, antiplatelet agents, and surgical interventions, have significantly improved clinical outcomes in patients with CVDs, major challenges persist. These include residual inflammatory risk, insufficient tissue repair after myocardial infarction, and in-stent restenosis [3]. Therefore, safer and more effective therapeutic strategies are urgently needed.

In recent years, plant-derived extracellular vesicles (PDEVs) have attracted increasing attention as potential candidates for cardiovascular applications because of their intrinsic bioactivity and amenability to engineering. PDEVs refer to nanoscale membrane-bound vesicles isolated from plant tissues or juices, typically ranging from 30 to 500 nm in diameter and containing diverse bioactive molecules, including nucleic acids, proteins, and secondary metabolites [4]. Their physicochemical properties and cargo profiles vary with plant species, tissue source, and preparation method, which may partly explain their heterogeneous biological effects [4]. Studies suggest that, in cardiovascular disease-related models, selected PDEVs can exert protective effects through anti-inflammatory and antioxidant activities, regulation of lipid metabolism, and promotion of endothelial repair [5,6]. Beyond their intrinsic bioactivity, PDEVs can support cargo loading and protect encapsulated agents, making them promising natural drug-delivery platforms [7]. Exposure of biological systems to exogenous materials can elicit immune responses. PDEVs’ compositional heterogeneity may pose theoretical cytotoxic and immunogenic risks; available evidence indicates that PDEVs exhibit low cytotoxicity, favorable biocompatibility, and no major adverse effects in vitro or in vivo [8,9]. Surface modification and stimulus-responsive design may further improve the delivery of PDEVs to injured endothelium, inflammatory cells, and ischemic tissues [10]. Nevertheless, systematic evaluations of PDEVs in cardiovascular disease remain limited.

Accordingly, this review is organized around the major pathological processes underlying cardiovascular diseases and summarizes recent advances in PDEVs research from therapeutic and translational perspectives. We first outline the biogenesis, isolation, and characterization of PDEVs. We then discuss the potential mechanisms by which PDEVs modulate inflammation and immune responses, oxidative stress, lipid metabolism, endothelial repair, vascular remodeling, and cell death. Finally, we examine PDEV engineering strategies, including cargo loading, surface targeting, stimulus-responsive delivery, and integration with biomaterials, and discuss the major challenges affecting their cardiovascular translation.

## 2. Overview of PDEVs

### 2.1. Biogenesis of PDEVs

**Figure 1:**
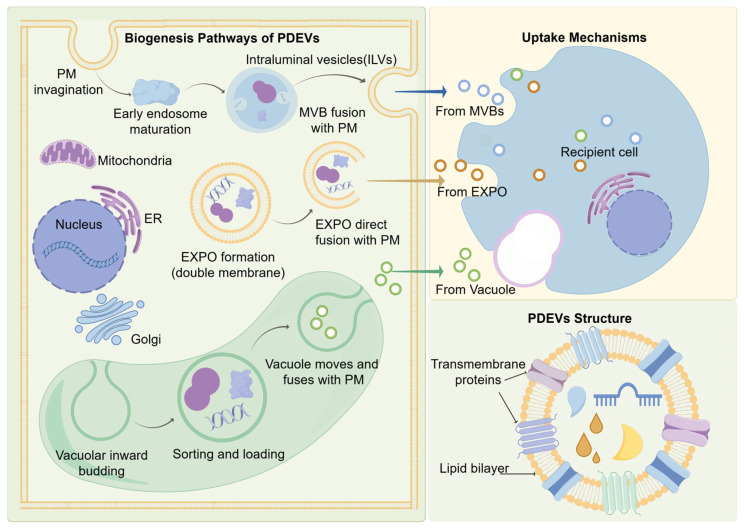
The biological occurrence pathway and structure of PDEVs.

The biogenesis of PDEVs is fundamental to understanding their compositional heterogeneity, functional diversity, and therapeutic potential. Current research suggests that PDEV release may involve at least three proposed pathways: the multivesicular body (MVB) pathway, the exocyst-positive organelle (EXPO) pathway, and the vacuolar pathway [6] (Figure 1). In the MVB pathway, early endosomes formed by plasma membrane invagination mature into MVBs after material exchange with the trans-Golgi network. Intraluminal vesicles (ILVs) then bud inward from the MVB membrane, selectively encapsulating bioactive cargos such as RNA, DNA, proteins, and lipids before being released as mature PDEVs [6,11]. In the EXPO pathway, the exocyst complex mediates the formation of a double-membrane EXPO at the plasma membrane, enabling direct extracellular transport of RNAs and functional proteins. In the vacuolar pathway, inward budding of the vacuolar membrane forms intraluminal vesicles that sort and package proteins, nucleic acids, and lipids; upon specific stimulation, the vacuole fuses with the plasma membrane and releases the enclosed vesicles into the extracellular space as PDEVs [6,11]. The released PDEVs are subsequently taken up by recipient cells. These pathways all involve membrane-bound organelles that produce lipid bilayer vesicles and selectively load RNAs, proteins, lipids, and plant secondary metabolites, thereby mediating material transport, intercellular communication, and stress responses [6].

Because of current technical limitations in isolation, most studies still analyze mixed vesicle populations derived from multiple biogenetic routes. Available evidence suggests that vesicles generated through different pathways differ in composition and function, potentially influencing their suitability for cardiovascular applications [12]. MVB-derived PDEVs are generally smaller (30–150 nm), and exhibit relatively favorable stability and tissue penetration, which may support long-distance intercellular communication and in vivo delivery [13]. In contrast, EXPO-derived PDEVs are typically larger (200–500 nm) and are mainly associated with cell wall remodeling, protein secretion, and stress responses, with possible enrichment of components involved in extracellular matrix regulation, proteolysis, and defense [14,15]. The vacuolar pathway is more commonly activated under plant stress conditions and may preferentially enrich defense-related proteins and secondary metabolites, such as flavonoids and alkaloids, thereby conferring potential anti-inflammatory and antioxidant advantages [16,17]. Direct links between specific pathways and defined cardiovascular effects remain unclear. Multi-omics profiling and functional validation are needed to establish the origins and cardiovascular functions of distinct PDEV subpopulations.

### 2.2. Composition of PDEVs

Lipids are major structural components of PDEV membranes, and increasing attention has focused on their distinctive lipid composition. Lipidomic studies have shown that PDEVs commonly contain phosphatidic acid (PA), phosphatidylethanolamine (PE), and phosphatidylcholine (PC), and their lipid profiles vary substantially among plant sources [18]. Such variation may influence vesicle stability, membrane fusion, cellular uptake, and tissue distribution. In particular, phospholipids and sphingolipids have been implicated in maintaining membrane integrity and modulating biodistribution and cellular internalization [19]. Ginger-derived EVs are enriched in PA and plant-specific galactolipids, which may contribute to their membrane stability and resistance in the gastrointestinal environment [20]. Grapefruit-derived EVs, which contain relatively high levels of PE and PC, have also been associated with efficient macrophage uptake [21]. Direct causal relationships between individual lipid species and these functional properties remain to be established.

Proteins are major molecular components of PDEVs and include membrane-associated, stress-responsive, trafficking-related proteins [22]. Annexins, a family of Ca^2+^-regulated membrane-binding proteins, have been identified in EVs derived from citrus extracellular fluids, sunflower seeds, and Arabidopsis thaliana leaves. These proteins are associated with plant responses to biotic and abiotic stress, including antioxidant defense and ROS signaling [23]. Membrane proteins may also facilitate cellular uptake, as trypsin-mediated removal of surface proteins from garlic-derived PDEVs markedly reduced vesicle internalization [24]. Heat stress proteins are another critical component. HSP60, HSP70, HSP80, and HSP90 have also been detected in multiple PDEVs, citrus, lemon, and grapefruit. Several studies have shown that the enrichment characteristics of such molecular chaperone proteins are closely related to the molecular mechanisms in which plants cope with environmental stress [25,26,27]. The patellar protein was identified in PDEVs of Arabidopsis and citrus plants. According to annotations in the UniProt database, these carrier proteins may be involved in membrane transport events during the cell division phase, particularly in the cell plate formation process [26,28]. Many of these functions are inferred from annotations or enrichment analyses and require direct experimental validation.

Current research on PDEV-associated nucleic acids has focused primarily on RNA, particularly small RNAs, whereas evidence regarding DNA remains limited. Various RNA species, including miRNAs, siRNAs, and tRNA-derived fragments, have been detected in PDEVs from different plant sources [29]. High-throughput small RNA sequencing has revealed source-dependent miRNA profiles that differ from those of the corresponding parental tissues, suggesting that certain RNAs may be selectively enriched in PDEVs [30]. RNase-protection assays and cellular uptake studies further indicate that some nucleic acids are protected by the vesicular membrane and can be delivered to recipient cells [30,31]. Arabidopsis-derived EVs have been shown to transfer small RNAs and mRNAs to fungal cells and modulate gene expression in the recipients [32]. In onion-derived EVs, 259 miRNAs were identified, and bioinformatic analyses suggested that 20 highly represented miRNAs may be associated with human cancer- and cardiovascular disease-related pathways [33]. Nevertheless, the capacity of plant-derived small RNAs to regulate mammalian gene expression across kingdoms remains controversial. Although selected studies suggest that plant RNAs may reach mammalian cells and exert sequence-dependent effects under specific experimental conditions, direct evidence that endogenous PDEV-associated RNAs are delivered to human cardiovascular cells at biologically relevant copy numbers is still lacking.

PDEVs also contain abundant secondary metabolites, which represent important contributors to their pharmacological activity [7]. These metabolites may act together with PDEVs’ associated lipids, proteins, and RNAs to influence multiple biological pathways. For example, polyphenols may modulate the cellular redox state in a manner that favors miRNA-mediated gene silencing, whereas certain phospholipid components may facilitate the intracellular release of secondary metabolites [30,34]. The diverse cargo composition of PDEVs may contribute to their broad biological effects, although the relative contribution of each cargo class remains unclear.

### 2.3. Isolation and Purification of PDEVs

**Figure 2:**
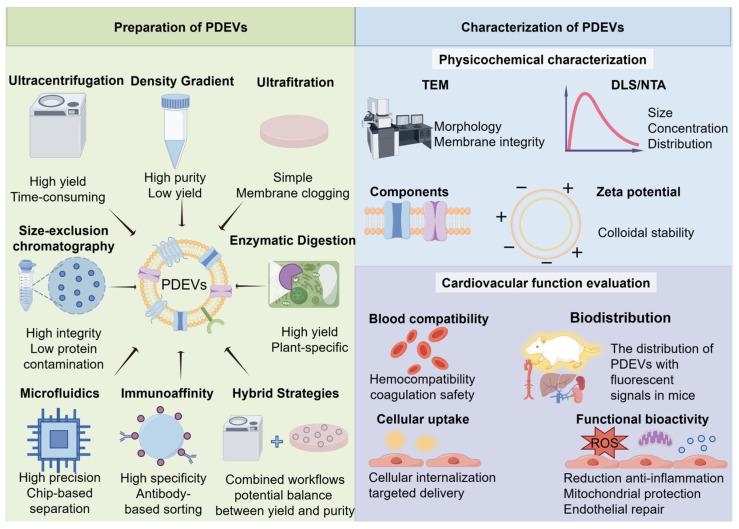
Preparation and Characterization of PDEVs.

High-quality isolation and purification are essential for both functional study and translational development of PDEVs. Commonly used methods include ultracentrifugation (UC), density-gradient centrifugation (DGC), polymer-based precipitation, ultrafiltration (UF), and size-exclusion chromatography (SEC) [35,36,37]. Emerging approaches include microfluidic separation and immunoaffinity capture, while enzymatic digestion may be used to facilitate vesicle release from plant tissues [35,36,37] (Figure 2). These methods differ substantially in purity, yield, preservation of vesicle integrity, and translational feasibility (Table 1), and such differences can directly influence the performance of PDEVs in cardiovascular studies.

Among these approaches, UC remains the most widely used because of its technical maturity, broad applicability, and relatively high yield. However, prolonged high-speed centrifugation may also cause vesicle aggregation, membrane damage, and co-isolation of impurities, thereby compromising size uniformity and biological activity [35]. Polymer precipitation, UF, and enzymatic digestion are relatively simple and efficient, but they often provide lower purity because proteins, polysaccharides, and other contaminants may be co-isolated. These methods are therefore more suitable for preliminary enrichment or exploratory large-scale preparation [36,37]. By contrast, DGC, SEC, microfluidics, and immunoaffinity capture generally offer higher purity and better preservation of vesicle structure and activity, making them more appropriate for mechanistic studies and in vivo evaluation. However, microfluidic and immunoaffinity-based methods usually require specialized equipment or reagents, which may limit their broader application [38,39].

In summary, no single method is optimal for all purposes. For cardiovascular applications, sequential workflows such as UC followed by DGC or SEC may offer a more favorable balance between particle recovery and purity while supporting vesicle integrity and biological activity, although their suitability should be validated for each plant source and intended use. Further optimization of standardized isolation workflows will be important for improving inter-study comparability and supporting the translational development of PDEVs.

### 2.4. Characterization of PDEVs

The physicochemical characterization of PDEVs is critical for both functional evaluation and translational development. Conventional characterization mainly includes transmission electron microscopy (TEM) for morphology, dynamic light scattering (DLS) and nanoparticle tracking analysis (NTA) for particle size, NTA for particle concentration, and electrophoretic light scattering for zeta potential [40,41].

In addition to these routine characterizations, cardiovascular research places greater emphasis on properties related to in vivo performance, including hemocompatibility, shear resistance, uptake by cardiovascular-relevant cells, and functional activity [42] (Figure 2). PDEVs with enriched phospholipid and sphingolipid content, and a stable negative surface charge are generally considered more favorable for circulatory stability and shear resistance [43,44]. Grapefruit-derived EVs showed no obvious hemolysis in a mouse model of vascular calcification [45]. Rehmannia-derived EVs were reported to be taken up by cardiomyoblasts and to reduce intracellular reactive oxygen species, suggesting a potential protective effect against oxidative stress [46]. Available biodistribution studies indicate that some PDEVs can accumulate at vascular lesions, while uptake is also detectable in the liver and spleen [45,47].

## 3. Applications and Mechanisms of PDEVs in Cardiovascular Diseases

**Figure 3:**
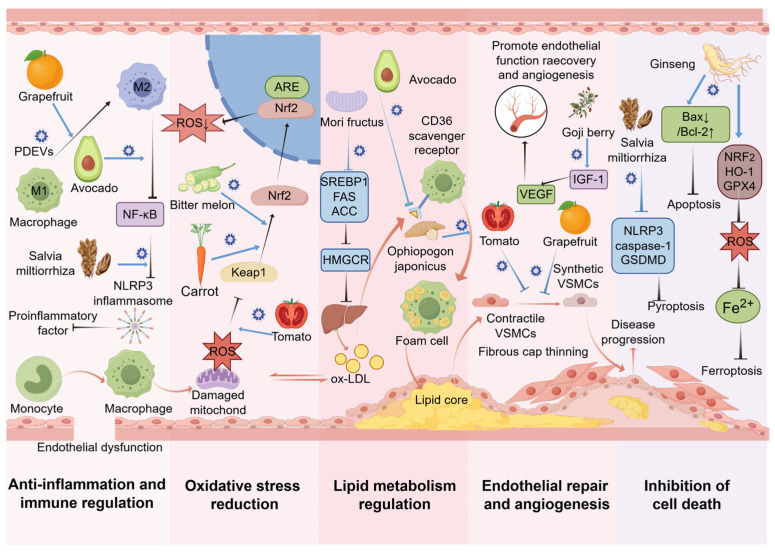
Mechanisms by which PDEVs may protect against cardiovascular diseases. Red arrows indicate pathological progression, whereas black and blue arrows indicate PDEV-mediated interventions. Upward and downward arrows indicate enhancement and inhibition, respectively.

Cardiovascular diseases are driven by multiple interacting pathological processes. Unlike single-target drugs, PDEVs contain diverse bioactive cargos and may therefore regulate disease progression through multiple targets and pathways [48]. Available preclinical evidence indicates that PDEVs exert cardiovascular protective effects by modulating inflammation and oxidative stress, improving lipid metabolism, preserving endothelial integrity, limiting vascular remodeling, and reducing pathological cell death [5] (Figure 3). These effects have been reported in experimental models of atherosclerosis, myocardial ischemia–reperfusion injury, myocardial infarction, vascular calcification, in-stent restenosis, and drug-induced cardiac injury (Table 2).

### 3.1. Anti-Inflammatory and Immune-Regulatory Effects

Chronic inflammation is a common pathological basis for the progression of many cardiovascular diseases [64]. The anti-inflammatory effects of PDEVs are mainly reflected in two aspects: the remodeling of immune cell phenotypes and the inhibition of pro-inflammatory signaling pathways. Macrophages are key effector cells in the cardiovascular inflammatory microenvironment, and an imbalance between the M1 and M2 phenotypes can markedly influence tissue injury, inflammation resolution, and repair [65]. Pueraria-derived PDEVs have been reported to promote macrophage polarization toward the M2 phenotype, thereby helping restore immune homeostasis in injured myocardium and vascular tissue [66].

In addition, NF-κB signaling and the NLRP3 inflammasome are two inflammatory axes that have received particular attention in studies of PDEVs. PDEVs derived from garlic and ginger have been shown to inhibit NLRP3 inflammasome activation and reduce the caspase-1-mediated maturation of IL-1β and IL-18 [67,68]. PDEVs derived from avocado, goji, and other plant sources can also modulate NF-κB activation and downregulate the expression of pro-inflammatory genes, including IL-6, IL-1β, COX-2, and iNOS, thereby attenuating endothelial inflammatory activation, monocyte adhesion, and local inflammatory responses [49,60]. PDEVs from different sources may act through distinct anti-inflammatory mechanisms. However, most existing studies remain limited to the observation of reduced inflammatory markers. Systematic evidence is still lacking on how PDEVs influence the spatiotemporal dynamics of immune cells, the resolution of inflammation, and subsequent tissue repair.

### 3.2. Regulation of Oxidative Stress

Anti-oxidative stress is an important mechanism underlying the cardiovascular protective effects of PDEVs. In pathological settings such as myocardial ischemia–reperfusion injury, drug-induced cardiotoxicity, radiation-induced cardiac injury, and restenosis after vascular injury, excessive production of reactive oxygen species can promote lipid peroxidation, DNA damage, and mitochondrial dysfunction, thereby impairing cardiomyocyte energy metabolism and survival [69]. Current evidence suggests that PDEVs may attenuate oxidative stress by reducing ROS levels, preserving mitochondrial function, and limiting cellular injury.

These effects may involve at least two mechanisms. First, PDEVs may enhance endogenous antioxidant defense pathways and thereby improve cellular resistance to oxidative stress. The Keap1/Nrf2/ARE pathway is a key regulator of redox homeostasis and mitochondrial function [70]. Tomato- and bitter melon-derived EVs have been reported to reduce KEAP1 expression, promote NRF2 nuclear translocation, and increase HO-1 and NQO1 expression, changes associated with reduced oxidative injury in models of post-injury restenosis and doxorubicin-induced cardiotoxicity, respectively [54,55].

Second, plant-derived bioactive components carried by PDEVs, including flavonoids, polyphenols, and lipids, may directly contribute to free radical scavenging and membrane stabilization. Citrus-derived EVs are enriched in flavonoids and have been shown to neutralize superoxide anions (O_2_^−^) and hydroxyl radicals, thereby reducing the cellular oxidative burden [71]. In addition to lowering overall oxidative stress, PDEVs may also help preserve mitochondrial function. For example, mulberry-derived EVs have been reported to reduce mitochondrial ROS in cardiomyocytes, restore mitochondrial membrane potential, and maintain ATP production, thereby attenuating cardiomyocyte injury [56]. These studies report reduced ROS levels and increased antioxidant-molecule expression after PDEVs treatment, but the responsible components remain undefined.

### 3.3. Regulation of Lipid Metabolism

Disordered lipid metabolism is a key process in the progression of atherosclerosis and vascular calcification. It involves excessive cholesterol synthesis, subendothelial lipid deposition, macrophage uptake of lipids with subsequent foam cell formation, and ultimately plaque development [72]. Current in vitro and in vivo evidence suggests that selected PDEV preparations may modulate multiple stages of this process. In terms of lipid synthesis, mulberry-derived PDEVs have been reported to target lipid synthesis-related genes, thereby reducing hepatic cholesterol synthesis, lowering blood lipid levels, and attenuating atherosclerotic lesions in ApoE−/− mice [57].

PDEVs also act during lipid uptake and foam cell formation. Berberis kaschgarica-derived EVs have been shown to reduce lipid deposition in the vascular endothelium and help maintain endothelial barrier function [58]. With respect to oxidized low-density lipoprotein (ox-LDL), which has crossed the endothelial barrier and undergone oxidative modification, onion-derived PDEVs can inhibit ox-LDL uptake by THP-1 macrophages and reduce foam cell formation [50]. Avocado-derived EVs loaded with ginkgetin and berberine have been reported to downregulate lipid scavenger receptors such as CD36, thereby further limiting lipid accumulation in macrophages [49].

At the stage of plaque stabilization, PDEVs may further contribute by improving the inflammatory microenvironment and matrix metabolism. Mulberry-derived EVs not only reduce blood lipid levels but also decrease plaque area [57]. Similarly, VHPK-engineered onion-derived EVs have been shown to suppress inflammation within plaques and reduce the secretion of matrix metalloproteinases (MMPs), thereby enhancing fibrous cap stability and lowering the risk of plaque rupture [50].

Overall, selected PDEVs may influence several processes involved in atherogenesis, including dyslipidemia, vascular lipid accumulation, macrophage foam-cell formation, and plaque-associated inflammation. Most studies have evaluated circulating lipid levels, foam-cell formation, or lesion area rather than direct features of plaque stability. Future studies should assess fibrous-cap structure, collagen content, necrotic-core size, plaque cellular composition, intraplaque hemorrhage, and treatment durability to determine whether the reported molecular and metabolic effects translate into stable plaque remodeling.

### 3.4. Endothelial Repair and Angiogenesis

Phenotypic switching of vascular smooth muscle cells (VSMCs) and endothelial cell (EC) dysfunction are key events in vascular repair and remodeling [73]. VSMC phenotypic transition is a common pathological basis for vascular calcification, aortic dissection, and restenosis after vascular injury [74]. Current preclinical evidence suggests that the protective effects of PDEVs on VSMCs are mainly related to their antioxidant activity and their ability to inhibit osteogenic transition [45,75]. In restenosis after vascular injury, tomato-derived EVs have been reported to reduce oxidative stress through the Keap1/Nrf2 axis and to suppress VSMC proliferation, migration, and matrix degradation [54]. In vascular calcification, grapefruit-derived EVs were shown to inhibit the ROS-AKT/Runx2 pathway, reduce RUNX2 and BMP2 expression, enhance antioxidant enzyme activity, and preserve α-SMA expression, thereby attenuating the osteogenic transition of VSMCs [45]. These effects may help maintain VSMCs in a more favorable contractile phenotype and support vascular patency. However, the role of VSMCs is context-dependent. Excessive proliferation and migration may promote restenosis at early stages, whereas in advanced plaques VSMCs contribute to fibrous cap formation and plaque stability [74,76]. Therefore, the effects of PDEVs on VSMCs should be evaluated not only by their inhibition of proliferation but also in relation to disease stage and vascular structural outcomes. There is currently no evidence that PDEVs regulate VSMCs differently according to plaque maturity, and their effects have not been directly compared among early, stable, and vulnerable plaques. Future studies should use stage-specific atherosclerosis models, VSMC lineage tracing, and assessments of fibrous-cap formation, collagen deposition, VSMC survival, and plaque vulnerability to determine whether the effects of PDEVs are stage-dependent and how they influence plaque stability.

In parallel, endothelial cells are essential for maintaining vascular homeostasis. Endothelial dysfunction may lead to impaired vasodilation, increased permeability, inflammatory cell adhesion, thrombosis, and microcirculatory disturbance [77]. Some PDEVs have been shown in both in vitro and in vivo studies to improve ischemic microenvironments by promoting endothelial cell survival, migration, and angiogenesis. For example, *Salvia miltiorrhiza*-derived EVs enhanced the viability and migratory capacity of human umbilical vein endothelial cells and promoted angiogenesis in ischemic myocardium [44]. Similarly, goji-derived EVs delivered in fibrin gel were reported to suppress the p38 MAPK/NF-κB pathway, reduce IL-1β, IL-6, and MMP-2 and MMP-9 expression, and upregulate VEGF-A, IGF-1, and CD31, thereby promoting vascular regeneration in infarcted myocardium [60]. This combined protective and reparative effect may be of particular interest in settings such as vascular stent implantation, where endothelial denudation and abnormal VSMC proliferation often coexist. In this context, PDEVs with both anti-restenotic and pro-endothelialization properties may have potential as stent-coating components.

Angiogenesis is not uniformly beneficial across all cardiovascular settings. In ischemic myocardium, it may improve oxygen supply and tissue repair, whereas in atherosclerotic plaques, excessive neovascularization may increase the risk of intraplaque hemorrhage and plaque instability [78,79]. Therefore, future studies should clarify whether PDEVs promote functional vascular regeneration in ischemic tissues or contribute to pathological neovascularization under certain conditions.

### 3.5. Inhibition of Cell Death

Abnormal death of cardiovascular cells is a key contributor to structural damage in the heart and vasculature and may ultimately promote the development of heart failure or myocardial infarction [80]. Under pathological conditions, apoptosis, pyroptosis, and ferroptosis often interact with one another and jointly contribute to inflammatory amplification, oxidative injury, and tissue remodeling [81]. In this context, some PDEVs appear to exert protective effects through multiple modes of cell death regulation.

Regarding cardiomyocyte apoptosis, PDEVs may act by preserving mitochondrial homeostasis and inhibiting stress-related signaling pathways. For example, carrot-derived EVs have been reported to increase the Bcl-2/Bax ratio and suppress caspase-3 activation, thereby attenuating H_2_O_2_-induced cardiomyocyte injury [53]. Ginseng-derived EVs were shown to reduce cisplatin-induced apoptosis through inhibition of the MAPK signaling pathway, including p38, JNK, and ERK [61]. With respect to pyroptosis, PDEVs appear to act mainly through the NLRP3 inflammasome. Garlic- and Chinese chive-derived EVs have been reported to interfere with NLRP3 assembly through their bioactive lipid components, thereby suppressing inflammasome-associated pyroptosis and potentially limiting acute inflammatory injury [67].

In the regulation of ferroptosis, evidence from mice suggests that PDEVs may act by enhancing antioxidant defense pathways. PDEVs derived from ginseng have been shown to activate the Nrf2/HO-1/GPX4 axis, reduce ROS, MDA, and Fe^2+^ accumulation, and thereby attenuate lipid peroxidation and ferroptotic injury [63]. However, evidence in this area remains limited. Future studies should further examine the dynamic changes and relative contribution of different cell death pathways within the same disease model.

## 4. Engineering Strategies of PDEVs

**Figure 4:**
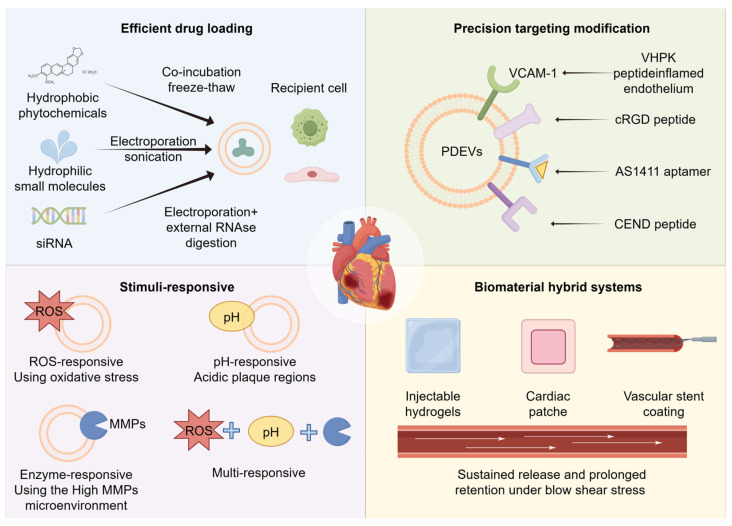
Engineering transformation strategies for PDEVs.

Natural PDEVs combine intrinsic bioactive cargo with a membrane-based nanostructure. However, their therapeutic application in cardiovascular diseases is still limited by modest efficacy, insufficient lesion targeting, clearance by the mononuclear phagocyte system, short circulation time, and limited stability under complex hemodynamic conditions [82]. In recent years, engineering has become an important strategy for improving their therapeutic performance. Current approaches mainly include drug loading, surface targeting modification, stimulus-responsive release, and combination with biomaterials [83] (Figure 4). These strategies are intended to address several key questions, namely what to load, where to deliver it, when to release it, and how to achieve prolonged local retention.

### 4.1. Drug Loading Strategies

PDEVs can take advantage of their natural lipid bilayer structure to load drugs with defined pharmacological activities. This helps improve solubility, bioavailability, and in vivo stability of these agents, while also enabling synergistic enhancement between carrier-mediated delivery and drug efficacy [84]. In cardiovascular research, commonly used loading methods include co-incubation, electroporation, ultrasound, and freeze–thaw cycles [85]. Previous studies have shown that ginkgetin and berberine can be loaded into avocado-derived PDEVs through co-incubation and freeze–thaw treatment. The resulting vesicles were effectively taken up by macrophages and inhibited foam cell formation and inflammatory cytokine expression, thereby exerting anti-atherosclerotic effects [49]. Feng used electroporation to load sodium thiosulfate into grapefruit-derived EVs, which significantly increased drug accumulation in calcified vascular regions and reduced vascular smooth muscle cell calcification and inflammation [45]. Wang reported that Coptis chinensis-derived EVs loaded with CA1-siRNA by electroporation combined with RNase treatment promoted endothelial repair, inhibited NF-κB signaling, and reduced neointimal formation in restenosis models [86].

Different types of therapeutic cargos may require different loading strategies. Hydrophobic bioactive compounds, such as ginkgetin and berberine, may be incorporated directly into the lipid bilayer of PDEVs through co-incubation or freeze–thaw treatment, thereby improving their in vivo stability and bioavailability. In contrast, hydrophilic small molecules such as sodium thiosulfate and nucleic acid-based therapeutics may be better introduced into vesicles by ultrasound or electroporation to enhance local accumulation at diseased sites. Importantly, the loading process itself may alter the size, surface charge, and membrane integrity of PDEVs. Therefore, loaded PDEVs should be evaluated not only for loading capacity and encapsulation efficiency, but also for structural stability, release kinetics, and cellular uptake, to ensure that improved therapeutic efficacy is not achieved at the expense of vesicle function.

### 4.2. Surface Targeting Modification

After systemic administration, natural PDEVs are readily taken up and sequestered by the mononuclear phagocyte system, particularly in the liver and spleen [87]. Surface targeting modification has therefore become an important strategy to improve the selective delivery of PDEVs to diseased sites. Common targeting approaches include the use of peptides, antibodies, and aptamers. Among these, the VHPK peptide is one of the studied ligands because it can recognize vascular cell adhesion molecule-1 (VCAM-1), which is highly expressed in injured vascular endothelium [88]. Choi showed that onion-derived EVs modified with VHPK peptide displayed increased accumulation in atherosclerotic plaques and enhanced uptake by inflamed endothelial cells and macrophages, thereby suppressing local inflammation and plaque formation [50].

These findings suggest that surface engineering may provide a feasible strategy for enhancing the targeted delivery of PDEVs to inflamed vascular lesions. Based on this concept, different targeting ligands may be selected according to specific features of cardiovascular lesions. For example, in vascular stenting and endothelial injury, cRGD peptides may be used to target integrins that are upregulated on injured vascular walls, whereas the AS1411 aptamer may be useful for recognizing nucleolin on abnormally proliferating vascular smooth muscle cells [89,90]. In myocardial ischemia, peptides such as CEND-1 and RGD may help direct PDEVs toward ischemic or angiogenic tissues [91,92]. In atherosclerosis and vascular calcification, in addition to VHPK peptide, ligands such as hyaluronic acid, folate, or anti-CD36 nanobodies may improve targeting to highly activated macrophages within lesions [93,94,95,96]. It should be noted that most of these targeting strategies remain at the conceptual or early experimental stage. Further studies are needed to determine whether surface modification can truly improve in vivo lesion accumulation and therapeutic efficacy, rather than simply increasing cellular uptake in vitro.

### 4.3. Stimulus-Responsive PDEVs-Based Systems

Cardiovascular lesions are often characterized by local acidosis, elevated ROS levels, and abnormal activation of MMPs, providing a basis for the design of stimulus-responsive PDEVs [97,98,99]. Compared with passive release, stimulus-responsive PDEVs may enable selective drug release within the lesion microenvironment, thereby improving local therapeutic efficacy and reducing off-target effects.

The ROS-responsive system is applicable to diseases characterized by high oxidative stress such as myocardial ischemia–reperfusion injury, atherosclerosis, and drug-induced cardiotoxicity [100,101]. By introducing ROS-sensitive structures such as thioacetals, selenocarbonyl bonds, or borate ester bonds, drug release in a high-ROS environment can be achieved, and it can form a synergistic effect with PDEVs derived from ginger, tea, and citrus fruits, which are rich in polyphenols, flavonoids, and antioxidant lipids [16,102]. For example, PDEVs from Citrus medica combined with ROS-responsive materials were reported to promote drug release under oxidative conditions and to facilitate the polarization of pro-inflammatory macrophages toward an anti-inflammatory phenotype, thereby alleviating transplant-related ischemia–reperfusion injury [103]. pH-responsive systems exploit the acidic microenvironment of inflammatory or ischemic lesions and may be more suitable for chronic inflammatory vascular diseases, atherosclerotic plaques, and ischemic tissues [104]. Enzyme-responsive systems, by contrast, can take advantage of the abnormal expression of proteases such as MMPs in plaques, post-infarction remodeling, and restenosis to achieve local release [105,106].

However, single-responsive systems may be affected by microenvironmental heterogeneity, resulting in unstable or nonspecific drug release [107]. Dual-responsive or multi-responsive PDEV-based delivery systems may be more suitable for precise delivery in complex cardiovascular lesions [108,109].

### 4.4. PDEVs and Biomaterial Composites

Free PDEVs have limited in vivo residence time and tend to accumulate in reticuloendothelial organs such as the liver and spleen [110]. Incorporating selected PDEVs into biomaterials such as hydrogels, scaffolds, or cardiac patches may prolong local retention and enable sustained release. This strategy may have potential value for conditions requiring prolonged local intervention, including myocardial infarction, ischemia–reperfusion injury, and restenosis following vascular injury [60,111]. Hydrogels are among the most commonly investigated biomaterials for local PDEV delivery. Their three-dimensional hydrated polymer networks can entrap EVs at the lesion site, reduce early dispersion, and prolong local retention and release [112]. Injectable or in situ-forming hydrogel systems may also conform to irregularly shaped myocardial lesions [113]. Preliminary benefits have been reported in mouse models of cardiovascular injury. Lycium barbarum-derived PDEVs incorporated into fibrin gel showed increased local retention at the infarct site and improved cardiac function and short-term survival in mice with myocardial infarction, while reducing inflammation and cardiomyocyte apoptosis and promoting vascular regeneration [60]. Similarly, *Salvia miltiorrhiza*-derived PDEVs delivered using a hydrogel exhibited antioxidant and anti-apoptotic effects and alleviated inflammatory myocardial injury in an LPS-induced mouse model [62]. These findings highlight the potential of hydrogel-based PDEV delivery; however, evidence in cardiovascular disease remains limited. Future studies should compare different combinations of plant sources and hydrogel materials and systematically evaluate the quality attributes and biological activity of PDEVs after release, as well as the biodegradability, biocompatibility, and overall safety of the resulting composite systems.

## 5. Challenges and Prospects for the Clinical Translation of PDEVs

**Figure 5:**
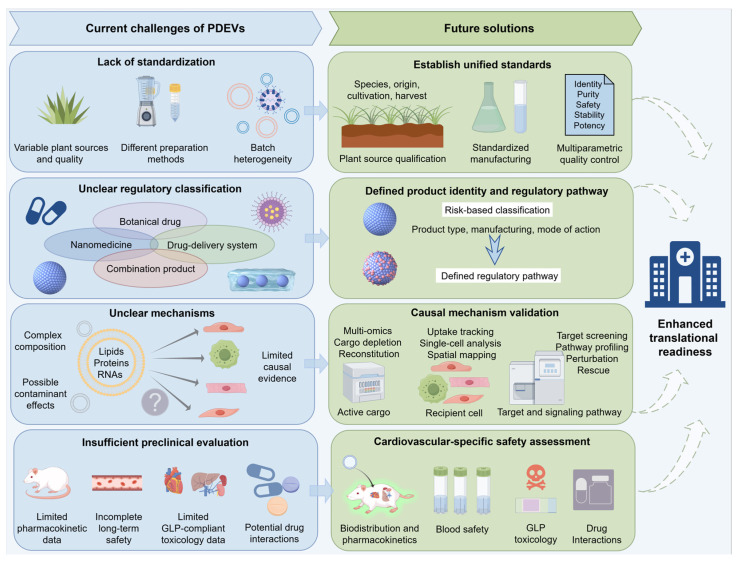
Challenges and solutions for Clinical Translation of PDEVs.

Available experimental evidence suggests that PDEVs have translational potential in cardiovascular diseases, but substantial challenges remain before clinical application (Figure 5).

### 5.1. Lack of Standardization

A major obstacle to the clinical translation of PDEVs is the absence of standardization. MISEV2023 provides a valuable general framework for reporting PDEV studies, but its coverage of PDEV-specific issues remains limited and should be further supplemented and refined [42]. Variations in raw materials and preparation methods can substantially alter PDEVs’ composition and contribute to batch-to-batch and inter-laboratory variability [41]. Moreover, PDEVs lack universally accepted PDEV-specific biomarkers, making characterization based solely on morphology, size, and zeta potential insufficient to establish their identity and purity or to distinguish them from co-isolated materials.

Standardization should encompass raw material qualification, manufacturing, and final-product characterization. First, raw material qualification criteria should be defined, including plant origin, cultivation and harvest conditions, post-harvest processing, storage, pesticide exposure, and microbial burden. For PDEVs derived from the same source, critical isolation, purification, and sterile processing parameters should be harmonized and adapted for scalable production. Minimum batch-release specifications should also be established for prepared PDEVs, including morphology, particle concentration, zeta potential, particle-size distribution, levels of contaminants, including endotoxins, pesticide residues, and allergens, as well as storage and freeze–thaw stability. In the absence of a universally accepted PDEV-specific marker, PDEV identity should be supported by multiple complementary analyses, including documentation of the plant source and sample-processing procedures, demonstration of membrane-bound vesicular structures, biophysical characterization, lipid and protein profiling, and assessment of sources or subpopulation-associated candidate markers [114]. Purity should be assessed using combined metrics, such as particle-to-protein ratio, membrane-bound particle enrichment, and residual co-isolated impurities [115]. In parallel, potency assays relevant to the intended cardiovascular application should be developed and validated to ensure functional consistency across batches.

### 5.2. Unclear Regulatory Classification

Regulatory classification is fundamental because it shapes the applicable chemistry, manufacturing, and controls framework, product testing and release specifications, nonclinical and pharmacokinetic programs, and clinical trial design. However, no unified regulatory framework has been established for PDEVs. It is necessary to distinguish between native and engineered products. Native PDEVs are compositionally heterogeneous and may contain co-isolated plant constituents, complicating the definition of product identity [26]. When their effects are mainly attributable to endogenous plant bioactive components, they may resemble botanical drugs; however, existing botanical-drug frameworks may not adequately address their vesicular structure and particle heterogeneity. Depending on their composition, manufacturing process, and mode of action, they may also be regulated as biological products or nanomedicines. Engineered PDEVs introduce additional complexity through cargo loading, surface modification, stimulus-responsive functionalization, or biomaterial integration. Such products may be regulated as drug-delivery systems or combination products. Regulatory pathways should adopt a risk-based, product-specific approach that considers plant source, active components, manufacturing process, degree of engineering, mode of action, and intended clinical use, while standardizing nomenclature and defining product boundaries [116,117].

### 5.3. Complex Composition and Unclear Mechanisms of Action

PDEVs contain diverse components, which may underlie their multi-target therapeutic potential but also make causal attribution between cargo and function a major challenge. Most current studies remain at the phenotypic level, showing that PDEV preparations alleviate specific pathological changes without systematically identifying the active components, molecular targets, or underlying mechanisms [22]. It also remains unclear whether some observed effects arise from contaminants co-isolated during preparation. Resolving these possibilities will require a stepwise mechanistic framework [118]. The requirement for vesicle integrity should first be examined by disrupting PDEV membranes and comparing the activities of intact and disrupted preparations. Comparisons with dose-matched free-compound controls can then determine whether vesicular encapsulation enhances bioavailability, whereas synthetic liposomes or inert nanoparticles matched for size, surface charge, and dose can help exclude nonspecific physicochemical effects. RNase, protease, or lipase treatment, together with selective depletion or enrichment of candidate cargo components, may identify the classes of components required for biological activity. Multi-omics profiling, single-cell sequencing, and spatial transcriptomics could further define the key cargo molecules, recipient cell populations, and molecular targets within cardiovascular tissues. Ultimately, depletion, reconstitution, and rescue experiments will be necessary to establish causal relationships among specific cargo components, molecular targets, and biological effects [118,119].

### 5.4. Incomplete Preclinical Evaluation

Preliminary preclinical evidence has accumulated regarding the biodistribution, hemocompatibility, administration feasibility, and short-term safety of PDEVs. Biodistribution studies have shown that intravenously administered 125I-labeled grapefruit EVs are widely distributed across multiple organs in mice [47], whereas hydroxyapatite-binding peptide-modified grapefruit EVs preferentially accumulate in calcified arteries in models of vascular calcification [45]. Orally administered cloudberry-derived nanovesicles have also been reported to exhibit prolonged gastrointestinal retention and effective systemic absorption [120]. In terms of hemocompatibility, engineered grapefruit EVs showed no evident hemolysis, while prickly pear-derived FicoVes likewise exhibited favorable compatibility [45,121]. Oral PDEVs can resist gastrointestinal digestion, retain structural integrity, and undergo efficient uptake and transepithelial transport across Caco-2 cell monolayers [120]; intravenous and intranasal administration have also been shown to be feasible [122]. Safety studies have reported no major toxicity for several PDEV preparations in vitro or in vivo, and early clinical studies involving grapefruit-, ginger-, turmeric-, and ginseng-derived PDEVs have indicated favorable short-term tolerability, with no evident immunogenicity or dose-limiting toxicity [119,123]. Nevertheless, an edible plant origin should not be equated with absolute safety, particularly under high-dose, repeated-dose, or non-oral administration conditions. Overall, although the available preclinical safety data for PDEVs are encouraging, they remain insufficient to support their direct clinical use in human cardiovascular therapy. A cardiovascular-specific preclinical safety evaluation framework should therefore be established.

This framework should quantitatively characterize biodistribution and pharmacokinetics, including circulation half-life, clearance, systemic exposure, and accumulation in cardiovascular lesions and major organs [19,124]. Hemocompatibility, thrombogenicity, complement activation, and immunogenicity should also be systematically assessed [123]. GLP-compliant toxicology studies should evaluate single- and repeated-dose toxicity, major-organ histopathology, local tolerance, and long-term safety to define safety margins and identify target-organ toxicity [123]. When plant-derived or exogenously loaded RNAs are proposed as active cargo, their stability, intracellular delivery, target specificity, innate immune activation, off-target effects, and potential genotoxicity-related risks require evaluation [125,126]. Finally, because cardiovascular patients are often elderly and receive multiple medications, potential interactions with antiplatelet agents, anticoagulants, statins, antihypertensive drugs, glucose-lowering therapies, and heart failure medications should be investigated.

## 6. Conclusions

In summary, current evidence suggests that PDEVs may have potential both as cardiovascular therapeutics and as drug-delivery platforms. Their capacity to modulate inflammation, oxidative stress, and lipid metabolism may support the simultaneous targeting of multiple pathological processes. However, most available evidence is derived from in vitro studies and small-animal models, whereas quantitative pharmacokinetic studies, long-term safety assessments, large-animal validation, and human clinical evidence remain insufficient. Additional challenges include the lack of standardized preparation and quality-control procedures and the incomplete understanding of cargo–function relationships. Future studies should investigate these issues in greater depth and, on the basis of established standardization, move beyond simply demonstrating efficacy to clarifying why PDEVs work, how their effects can be reproduced consistently, and how they can be translated safely.

## Figures and Tables

**Table 1:** Advantages and limitations for PDEVs isolation and purification [35,36,37].

Isolation Method	Principle	Advantages	Limitations	Suitable Applications
Ultracentrifugation	Differences in particle sedimentation coefficients	Technically mature, simple to operate, widely used	Requires specialized equipment, time-consuming, may cause vesicle membrane damage, co-precipitates cellular debris	Preliminary isolation and enrichment
Density-gradient centrifugation	Differences in buoyant density	High purity, relatively low recovery	Time-consuming, technically complex, low throughput, requires removal of the gradient medium	Mechanistic studies
Ultrafiltration	Size-based separation using membranes with different pore sizes	Rapid and inexpensive	Relatively low purity, membrane fouling, potential damage to vesicle structure	Preliminary concentration of large-volume samples
Size-exclusion chromatography	Differences in particle retention time within porous matrices	Preserves membrane integrity and biological activity, relatively high purity	Time-consuming, technically demanding, sample dilution, limited column capacity	Functional and in vivo studies
Polymer-based precipitation	Polymers reduce vesicle solubility and induce precipitation	Simple to operate, high processing capacity and recovery, low cost	Residual reagents may interfere with downstream analyses, co-precipitation of other contaminants	Rapid preliminary enrichment and exploratory screening of large-volume samples
Enzymatic cell-wall digestion	Enzymatic degradation of plant cell walls	Increase vesicle release from specific plant tissues, preserving structural integrity	May alter surface proteins, membrane structure, and cargo, potential enzyme residues	Pretreatment of plant tissues and studies aimed at improving vesicle extraction efficiency
Microfluidic separation	Differences in particle Electrophoretic mobility and refractive index	Rapid, preserves vesicle integrity, high reproducibility, automation	High equipment cost, susceptibility to clogging, low throughput	Small-scale analyses and mechanistic studies
Immunomagnetic bead capture	Specific antigen–antibody interactions	High specificity and purity	Dependence on marker expression, high cost	Studies of specific subpopulations

**Table 2:** Studies of PDEVs in cardiovascular diseases.

Category	PDEVs Source	Study Type and Experimental Model	Route and Dose	Disease	Potential Effects
Anti-inflammatory and immunomodulatory	Avocado + ginkgetin + berberine [49]	In vitro: LPS-induced macrophage inflammation model	In vitro: 1 mg/1 × 10^6^ cells	Atherosclerosis	Reduced expression of inflammatory mediators
	Onion + VHPK peptide [50]	In vitro: LPS-stimulated HUVEC and THP-1 inflammation models	In vitro: 1 × 10^10^ particles/mL	Atherosclerosis	Attenuated HUVEC inflammation and monocyte adhesion; reduced serum inflammatory mediators
	*Carthamus tinctorius* L. [51]	In vitro: ox-LDL-induced HUVEC injury; in vivo: high-fat diet-fed ApoE−/− mice	In vitro: not specified; in vivo: oral gavage, 40 mg/kg	Atherosclerosis	Reduced the release of inflammatory mediators
	Grapefruit + HA-binding peptide [45]	In vitro: high-phosphate-induced VSMC calcification; in vivo: cholecalciferol-induced vascular calcification in mice	In vitro: 5 mg/L; in vivo: intraperitoneal injection, 1.58 mg/kg	Vascular calcification	Reduced expression of inflammatory mediators
	*Salvia miltiorrhiza* [52]	In vitro: LPS + ATP-stimulated RAW264.7 macrophages; in vivo: high-fat diet/STZ-induced diabetic cardiomyopathy in C57BL/6 mice	In vitro: 100 μg/mL; in vivo: tail-vein injection, 10 mg/kg	Diabetic cardiomyopathy	Reduced expression of inflammatory mediators; improved glucose and lipid metabolism
Antioxidant	Carrot [53]	In vitro: H_2_O_2_-induced oxidative stress injury in H9C2 cells	In vitro: 1 × 10^11^ particles/mL	Myocardial oxidative injury associated with myocardial infarction	Attenuated oxidative stress-induced cellular injury
	Tomato [54]	In vitro: PDGF-BB-induced VSMC proliferation, migration, and phenotypic switching; in vivo: wire injury of the mouse carotid artery	In vitro: 100 μg/mL; in vivo: oral gavage, 1 mg/kg	Post-injury restenosis	Inhibited VSMC proliferation, migration, and phenotypic switching; reduced carotid neointimal area
	*Bitter melon* [55]	In vitro: doxorubicin (DOX)-induced injury in H9c2 cells and neonatal rat ventricular myocytes; in vivo: DOX-treated SD rats and C57BL/6J mice	In vitro: 10 μg/mL; in vivo: intravenous injection, 800 μg/kg	Cardiotoxicity	Restored mitochondrial function and cellular redox homeostasis
	*Bitter melon* [56]	In vitro: 16 Gy-irradiated H9C2 cells; in vivo: X-ray-induced cardiac injury in BALB/c nude mice	In vitro: 10 μg/mL; in vivo: intraperitoneal injection, 100 μg/kg	Cardiac injury	Restored mitochondrial function and cellular redox homeostasis
Lipid regulation and anti-atherosclerotic activity	Avocado + ginkgetin + berberine [49]	In vitro: LPS-induced macrophage inflammation model	In vitro: 1 mg/1 × 10^6^ cells	Atherosclerosis	Inhibited macrophage foam-cell formation
	Onion + VHPK peptide [50]	In vitro: LPS-stimulated HUVEC and THP-1 inflammation models	In vitro: 1 × 10^10^ particles/mL	Atherosclerosis	Reduced aortic plaque area
	*Mori fructus* [57]	In vitro: FFA-induced lipid accumulation in HepG2 cells; in vivo: high-fat diet-induced atherosclerosis in ApoE−/− mice	In vitro: 10 μg/mL; in vivo: intraperitoneal injection, 5 or 50 μg/mouse	Atherosclerosis	Improved lipid metabolism; reduced aortic plaque area; enhanced plaque stability
	*Berberis kashgarica* Rupr. [58]	In vitro: palmitic acid-induced lipid deposition in HUVECs	Not specified	Atherosclerosis	Improved lipid metabolism; reduced lipid deposition in HUVECs
	*Ophiopogon japonicus* [59]	In vitro: ox-LDL-induced foam-cell formation in THP-1 macrophages	In vitro: 1 × 10^10^ particles/mL	Myocardial infarction	Inhibited ox-LDL uptake
Endothelial protection and angiogenesis	Grapefruit + HA-binding peptide [45]	In vitro: high-phosphate-induced VSMC calcification; in vivo: cholecalciferol-induced vascular calcification in mice	In vitro: 5 mg/L; in vivo: intraperitoneal injection, 1.58 mg/kg	Vascular calcification	Reduced inflammatory mediator expression and aortic calcification area
	Tomato [54]	In vitro: PDGF-BB-induced VSMC proliferation, migration, and phenotypic switching; in vivo: wire injury of the mouse carotid artery	In vitro: 100 μg/mL; in vivo: oral gavage, 1 mg/kg	Post-injury restenosis	Inhibited VSMC proliferation, migration, and phenotypic switching; reduced carotid neointimal area
	*Ophiopogon japonicus* [59]	In vitro: ox-LDL-induced foam-cell formation in THP-1 macrophages	In vitro: 1 × 10^10^ particles/mL	Myocardial infarction	Reduced high-glucose-induced HUVEC apoptosis; promoted migration and tube formation
	Gouqi + fibringel [60]	In vitro: hypoxic HL-1 cells; in vivo: left anterior descending coronary artery ligation in C57BL/6 mice	In vitro: 5 × 10^7^ particles/mL; in vivo: local myocardial gel application, 1 × 10^8^ particles/mouse	Myocardial infarction	Improved 14-day survival and cardiac function; reduced infarct size and fibrosis; promoted angiogenesis
	*Salvia miltiorrhiza* [44]	In vitro: HUVECs; in vivo: left anterior descending coronary artery ligation in C57BL/6 mice	In vitro: 100 μg/mL; in vivo: tail-vein injection, 10 mg/kg	Myocardial ischemia–reperfusion injury	Promoted HUVEC proliferation and migration; enhanced angiogenesis
Anti-apoptotic	Carrot [53]	In vitro: H_2_O_2_-induced oxidative stress injury in H9C2 cells	In vitro: 1 × 10^11^ particles/mL	Myocardial oxidative injury associated with myocardial infarction	Attenuated oxidative stress-induced cellular injury and inhibited apoptosis
	Gouqi + fibringel [60]	In vitro: hypoxic HL-1 cells; in vivo: permanent LAD ligation in C57BL/6 mice	In vitro: 5 × 10^7^ particles/mL; in vivo: local myocardial gel application, 1 × 10^8^ particles/mouse	Myocardial infarction	Improved 14-day survival and cardiac function; reduced infarct size and fibrosis
	Ginseng [61]	In vitro: cisplatin-treated H9C2 cells; in vivo: cisplatin-induced cardiotoxicity in ICR mice	In vitro: 40 μg/mL; in vivo: 150 or 300 μg/mouse	Cardiotoxicity	Improved lipid metabolism and attenuated cardiac tissue injury
	*Salvia miltiorrhiza* + thermosensitive hydrogel [62]	In vitro: H_2_O_2_-induced H9C2 injury; in vivo: LPS-induced myocardial injury in C57BL/6 mice	In vitro: 10 μg/mL; in vivo: local intramyocardial injection, dose not reported	Myocardial injury	Reduced cardiomyocyte apoptosis; improved cardiac function; attenuated myocardial structural damage
Anti-pyroptotic	*Salvia miltiorrhiza* [52]	In vitro: LPS + ATP-stimulated RAW264.7 macrophages; in vivo: high-fat diet/STZ-induced diabetic cardiomyopathy in C57BL/6 mice	In vitro: 100 μg/mL; in vivo: tail-vein injection, 10 mg/kg	Diabetic cardiomyopathy	Reduced myocardial fibrosis; improved glucose and lipid metabolism; inhibited macrophage pyroptosis
Anti-ferroptotic	*American ginseng* [63]	In vitro: DOX-injured H9C2 cells; in vivo: DOX-induced cardiotoxicity in C57BL/6 mice and zebrafish	In vitro: 30 μg/mL; in vivo: oral gavage, 1 × 10^10^ or 2 × 10^10^ particles/mL	Cardiotoxicity	Improved lipid metabolism and cardiac function

## Data Availability

No new data were created or analyzed in this study. Data sharing is not applicable to this article.

## Abbreviations

The following abbreviations are used in this manuscript:

CVDs	Cardiovascular diseases
PDEVs	Plant-derived extracellular vesicles
MVB	Multivesicular body
EXPO	Exocyst-positive organelle
TGN	Trans-Golgi network
ILVs	Intraluminal vesicles
UC	ultracentrifugation
DGC	density gradient centrifugation
UF	ultrafiltration
SEC	size-exclusion chromatography
TEM	transmission electron microscopy
DLS	dynamic light scattering
NTA	nanoparticle tracking analysis
ROS	reactive oxygen species
MMPs	matrix metalloproteinases
VSMCs	vascular smooth muscle cells
EC	endothelial cell
VCAM-1	vascular cell adhesion molecule-1
PA	Phosphatidic acid
PE	Phosphatidylethanolamine
PC	Phosphatidylcholine
EVs	Extracellular vesicles
ox-LDL	Oxidized low-density lipoprotein

## References

[1] Chong B., Jayabaskaran J., Jauhari S.M., Chan S.P., Goh R., Kueh M., Li H., Chin Y.H., Kong G., Anand V.V. (2025). Global burden of cardiovascular diseases: Projections from 2025 to 2050. Eur. J. Prev. Cardiol..

[2] Frąk W., Wojtasińska A., Lisińska W., Młynarska E., Franczyk B., Rysz J. (2022). Pathophysiology of cardiovascular diseases: New insights into molecular mechanisms of atherosclerosis, arterial hypertension, and coronary artery disease. Biomedicines.

[3] Narkhede M., Pardeshi A., Bhagat R., Dharme G. (2024). Review on Emerging Therapeutic Strategies for Managing Cardiovascular Disease. Curr. Cardiol. Rev..

[4] Wang Y., Wang J., Ma J., Zhou Y., Lu R. (2022). Focusing on Future Applications and Current Challenges of Plant Derived Extracellular Vesicles. Pharmaceuticals.

[5] Liu Y., Wang Y., Fang M. (2026). Bioengineering Applications of Chinese Herbal Medicine-Derived Exosomes in Cardiovascular Diseases: Mechanisms and Translational Prospects. Drug Des. Dev. Ther..

[6] Zuo Y., Zhang J., Sun B., Wang X., Wang R., Tian S., Miao M. (2025). A New Perspective on Regenerative Medicine: Plant-Derived Extracellular Vesicles. Biomolecules.

[7] Zhao B., Lin H., Jiang X., Li W., Gao Y., Li M., Yu Y., Chen N., Gao J. (2024). Exosome-like nanoparticles derived from fruits, vegetables, and herbs: Innovative strategies of therapeutic and drug delivery. Theranostics.

[8] Zeng H., Wang Q., Wang F., Tian Y., Xie Y., Dai D., Meng Y., Zou C., Wei L., He X. (2026). Extracellular vesicles derived from Citrus plants: Innovative applications in therapeutics and drug delivery. J. Nanobiotechnol..

[9] Sun Y., Liu Y., Li J., Huang S., Du Y., Chen D., Yang M., Peng Y. (2025). Taraxacum mongolicum Hand.-Mazz. derived extracellular vesicles alleviate mastitis via NLRP3 inflammasome and NF-κB/MAPK pathways. J. Mater. Chem. B.

[10] Li L., Wang F., Zhu D., Hu S., Cheng K., Li Z. (2025). Engineering exosomes and exosome-like nanovesicles for improving tissue targeting and retention. Fundam. Res..

[11] Xia X., Zhu J., Xu X., Wang W., Zhao R., Wu K., Huang H., Qian Y., Luo Z., Xu F. (2025). Current progress of plant-derived exosome-like nanovesicles on the regulation of osteoporosis and osteoarthritis. Ann. Med..

[12] Lawson C., Vicencio J.M., Yellon D.M., Davidson S.M. (2016). Microvesicles and exosomes: New players in metabolic and cardiovascular disease. J. Endocrinol..

[13] Li X., Bao H., Wang Z., Wang M., Fan B., Zhu C., Chen Z. (2018). Biogenesis and Function of Multivesicular Bodies in Plant Immunity. Front. Plant Sci..

[14] Wang J., Ding Y., Wang J., Hillmer S., Miao Y., Lo S.W., Wang X., Robinson D.G., Jiang L. (2010). EXPO, an exocyst-positive organelle distinct from multivesicular endosomes and autophagosomes, mediates cytosol to cell wall exocytosis in Arabidopsis and tobacco cells. Plant Cell.

[15] Gao J., Li Y., Zhang S., He Y., Liu Z., Wang J., Hua J., Chen J., Zhong J., Zhong H. (2026). Structural and Functional Characterization of EXPO-Derived Extracellular Vesicles in Plants. Adv. Sci..

[16] Kim M., Jang H., Kim W., Kim D., Park J.H. (2023). Therapeutic Applications of Plant-Derived Extracellular Vesicles as Antioxidants for Oxidative Stress-Related Diseases. Antioxidants.

[17] Farley J.T., Eldahshoury M.K., de Marcos Lousa C. (2022). Unconventional Secretion of Plant Extracellular Vesicles and Their Benefits to Human Health: A Mini Review. Front. Cell Dev. Biol..

[18] Wang F., Li L., Deng J., Ai J., Mo S., Ding D., Xiao Y., Hu S., Zhu D., Li Q. (2025). Lipidomic analysis of plant-derived extracellular vesicles for guidance of potential anti-cancer therapy. Bioact. Mater..

[19] Feng J., Xiu Q., Huang Y., Troyer Z., Li B., Zheng L. (2023). Plant-Derived Vesicle-like Nanoparticles as Promising Biotherapeutic Tools: Present and Future. Adv. Mater..

[20] Kumar A., Sundaram K., Teng Y., Mu J., Sriwastva M.K., Zhang L., Hood J.L., Yan J., Zhang X., Park J.W. (2022). Ginger nanoparticles mediated induction of Foxa2 prevents high-fat diet-induced insulin resistance. Theranostics.

[21] Wang B., Zhuang X., Deng Z.B., Jiang H., Mu J., Wang Q., Xiang X., Guo H., Zhang L., Dryden G. (2014). Targeted drug delivery to intestinal macrophages by bioactive nanovesicles released from grapefruit. Mol. Ther..

[22] Huang D., Chen J., Zhao M., Shen H., Jin Q., Xiao D., Peng Z., Chen T., Zhang Y., Rao D. (2025). Plant-derived extracellular vesicles: Composition, function and clinical potential. J. Transl. Med..

[23] Rutter B.D., Innes R.W. (2017). Extracellular Vesicles Isolated from the Leaf Apoplast Carry Stress-Response Proteins. Plant Physiol..

[24] Song H., Canup B., Ngo V.L., Denning T.L., Garg P., Laroui H. (2020). Internalization of Garlic-Derived Nanovesicles on Liver Cells is Triggered by Interaction with CD98. ACS Omega.

[25] Raimondo S., Naselli F., Fontana S., Monteleone F., Lo Dico A., Saieva L., Zito G., Flugy A., Manno M., Di Bella M.A. (2015). Citrus limon-derived nanovesicles inhibit cancer cell proliferation and suppress CML xenograft growth by inducing TRAIL-mediated cell death. Oncotarget.

[26] Pocsfalvi G., Turiák L., Ambrosone A., Del Gaudio P., Puska G., Fiume I., Silvestre T., Vékey K. (2018). Protein biocargo of citrus fruit-derived vesicles reveals heterogeneous transport and extracellular vesicle populations. J. Plant Physiol..

[27] Kim S.Q., Kim K.H. (2022). Emergence of Edible Plant-Derived Nanovesicles as Functional Food Components and Nanocarriers for Therapeutics Delivery: Potentials in Human Health and Disease. Cells.

[28] Regente M., Pinedo M., San Clemente H., Balliau T., Jamet E., de la Canal L. (2017). Plant extracellular vesicles are incorporated by a fungal pathogen and inhibit its growth. J. Exp. Bot..

[29] Tembo K.M., Wang X., Bolideei M., Liu Q., Baboni F., Mehran M.J., Sun F., Wang C.Y. (2025). Exploring the bioactivity of MicroRNAs Originated from Plant-derived Exosome-like Nanoparticles (PELNs): Current perspectives. J. Nanobiotechnol..

[30] López de Las Hazas M.C., Tomé-Carneiro J., Del Pozo-Acebo L., Del Saz-Lara A., Chapado L.A., Balaguer L., Rojo E., Espín J.C., Crespo C., Moreno D.A. (2023). Therapeutic potential of plant-derived extracellular vesicles as nanocarriers for exogenous miRNAs. Pharmacol. Res..

[31] AFawzy M.P., Hassan H.A.F.M., Amin M.U., Preis E., Bakowsky U., Fahmy S.A. (2025). Deploying nucleic acids-loaded plant-derived exosomes as green nano gadget in cancer gene therapy. Mater. Adv..

[32] Cai Q., Qiao L., Wang M., He B., Lin F.M., Palmquist J., Huang S.D., Jin H. (2018). Plants send small RNAs in extracellular vesicles to fungal pathogen to silence virulence genes. Science.

[33] Liang Z., Gu Y., Song J., Yang W., Xu X. (2026). Identification and analysis of small RNAs in exosome-like nanoparticles derived from onion (*Allium cepa* L.). Biochem. Biophys. Rep..

[34] Yang T., He M., Huang J., Zhang D., Song T., Tan J., Wang X., Lu Y., Kong Q., Zhang J. (2025). From Garden to Clinic: Plant-Derived Exosome-like Nanovesicles for Precision Oxidative Stress Therapy. Int. J. Nanomed..

[35] Yi Q., Xu Z., Thakur A., Zhang K., Liang Q., Liu Y., Yan Y. (2023). Current understanding of plant-derived exosome-like nanoparticles in regulating the inflammatory response and immune system microenvironment. Pharmacol. Res..

[36] Huang Y., Wang S., Cai Q., Jin H. (2021). Effective methods for isolation and purification of extracellular vesicles from plants. J. Integr. Plant Biol..

[37] Zheng M., Hong X., Liao P., Huang H., Zhang Y., Xu W., Li H. (2025). Plant-Derived Exosome-like Nanoparticles: A Promising Therapeutic for Neurological Disorders and Drug Delivery. Int. J. Nanomed..

[38] Li B., Wu W., Xu W., Qian H., Ji C. (2025). Advances of extracellular vesicles isolation and detection frontier technology: From heterogeneity analysis to clinical application. J. Nanobiotechnol..

[39] Ming T., Yang Y., Zhu J., Lin J., Yang W., Yu G., Wang P., Zhang E., Chen Q., Liu J. (2025). Ginger-Derived Exosome-like Nanoparticles: The Effect of Extraction Methods on Metabolites and in vitro Anti-Lung Cancer Activity. Int. J. Nanomed..

[40] Ma Y., Yang K., Hu H., Fu W., Li C., Zeng Y., Li X., Wang Y. (2025). Comparative Analysis of the Composition of Exosome-like Nanoparticles from Dried and Fresh *Portulaca oleracea* L. Molecules.

[41] Shu F., Hu Y., Sarsaiya S., Jin L., Yang X., Liu F., Zhu J., Chen G., Chen J. (2025). Plant-derived extracellular vesicles quality control: Key process progress and future research directions. Discov. Nano.

[42] Welsh J.A., Goberdhan D., O’Driscoll L., Buzas E.I., Blenkiron C., Bussolati B., Cai H., Di Vizio D., Driedonks T., Erdbrügger U. (2024). Minimal information for studies of extracellular vesicles (MISEV2023): From basic to advanced approaches. J. Extracell. Vesicles.

[43] Woodhouse F.G., Goldstein R.E. (2012). Shear-driven circulation patterns in lipid membrane vesicles. J. Fluid Mech..

[44] Zhang S., Xia J., Zhu Y., Dong M., Wang J. (2024). Establishing Salvia miltiorrhiza-Derived Exosome-like Nanoparticles and Elucidating Their Role in Angiogenesis. Molecules.

[45] Feng W., Teng Y., Zhong Q., Zhang Y., Zhang J., Zhao P., Chen G., Wang C., Liang X.J., Ou C. (2023). Biomimetic Grapefruit-Derived Extracellular Vesicles for Safe and Targeted Delivery of Sodium Thiosulfate against Vascular Calcification. ACS Nano.

[46] Fan H., Zhao S., Di Y., Yu Y., Sun Y., Wang Y., Li C., Wang J. (2026). *Rehmannia glutinosa* nanovesicles protect cardiomyoblasts from oxidative injury. Extracell. Vesicles Circ. Nucleic Acids.

[47] Garaeva L., Kamyshinsky R., Kil Y., Varfolomeeva E., Verlov N., Komarova E., Garmay Y., Landa S., Burdakov V., Myasnikov A. (2021). Delivery of functional exogenous proteins by plant-derived vesicles to human cells in vitro. Sci. Rep..

[48] Urzì O., Gasparro R., Ganji N.R., Alessandro R., Raimondo S. (2022). Plant-RNA in Extracellular Vesicles: The Secret of Cross-Kingdom Communication. Membranes.

[49] Sharma S., Mahanty M., Rahaman S.G., Mukherjee P., Dutta B., Khan M.I., Sankaran K.R., He X., Kesavalu L., Li W. (2024). Avocado-derived extracellular vesicles loaded with ginkgetin and berberine prevent inflammation and macrophage foam cell formation. J. Cell. Mol. Med..

[50] Choi C., Rhee W.J. (2025). Targeted Atherosclerosis Treatment Using Vascular Cell Adhesion Molecule-1 Targeting Peptide-Engineered Plant-Derived Extracellular Vesicles. Int. J. Mol. Sci..

[51] Yang R., Lin F., Wang W., Dai G., Ke X., Wu G. (2024). Investigating the therapeutic effects and mechanisms of *Carthamus tinctorius* L.-derived nanovesicles in atherosclerosis treatment. Cell Commun. Signal..

[52] Peng Z., Gong Z., Wang Z., Deng B., Zhang X., Lin J. (2025). Salvia miltiorrhiza-derived exosome-like nanoparticles improve diabetic cardiomyopathy by inhibiting NLRP3 inflammasome-mediated macrophage pyroptosis via targeting the NEDD4/SGK1 axis. Nanomedicine.

[53] Kim D.K., Rhee W.J. (2021). Antioxidative Effects of Carrot-Derived Nanovesicles in Cardiomyoblast and Neuroblastoma Cells. Pharmaceutics.

[54] Shen H., Zhang M., Liu D., Liang X., Chang Y., Hu X., Gao W. (2025). Solanum lycopersicum derived exosome-like nanovesicles alleviate restenosis after vascular injury through the Keap1/Nrf2 pathway. Food Funct..

[55] Ye C., Yan C., Bian S.J., Li X.R., Li Y., Wang K.X., Zhu Y.H., Wang L., Wang Y.C., Wang Y.Y. (2024). *Momordica charantia* L.-derived exosome-like nanovesicles stabilize p62 expression to ameliorate doxorubicin cardiotoxicity. J. Nanobiotechnol..

[56] Cui W.W., Ye C., Wang K.X., Yang X., Zhu P.Y., Hu K., Lan T., Huang L.Y., Wang W., Gu B. (2022). Momordica. charantia-Derived Extracellular Vesicles-like Nanovesicles Protect Cardiomyocytes Against Radiation Injury via Attenuating DNA Damage and Mitochondria Dysfunction. Front. Cardiovasc. Med..

[57] Sui Y., Sun X., Liu Q., Qi G., Yang Y., Zhang X., Francis O.B., Wang Y., Liu R., Li X. (2025). Mori fructus-derived extracellular vesicle-like nanoparticles regulate dyslipidemia and prevent atherosclerosis progression via miRNAs. Phytomedicine.

[58] Dilimulati D., Nueraihemaiti N., Baishan A., Hailati S., Aikebaier A., Paerhati Y., Zhou W. (2025). Isolation and Bioactive Characterization of Berberis kaschgarica Rupr-Derived Exosome-like Nanovesicles: Exploring Therapeutic Potential in Atherosclerosis Pathogenesis. Biology.

[59] Pan Y., Tan M., Li X., Chen Q., Tao J., Lin X., Zeng Z., Song Y., Jiang F. (2026). Ophiopogon japonicus-derived plant exosomes: A promising strategy for blocking the progression of diabetic vasculopathy to myocardial infarction. BMC Complement. Med. Ther..

[60] Zhou H.H., Zhou X., Pei J., Xu S., Jin B., Chen J., Zhang Z., Tang M., Liu Y., Nüssler A.K. (2025). A fibrin gel-loaded Gouqi-derived nanovesicle (GqDNV) repairs the heart after myocardial infarction by inhibiting p38 MAPK/NF-κB p65 pathway. J. Nanobiotechnol..

[61] Yang S., Guo J., Chen D., Sun Z., Pu L., Sun G., Yang M., Peng Y. (2025). The Cardioprotective Effect of Ginseng Derived Exosomes via Inhibition of Oxidative Stress and Apoptosis. ACS Appl. Bio Mater..

[62] Liu Z., Chen S., Dou B., Shen Z., Liu H., Wang X., Zhu L., Qiu Y., Lyu Z., Ba T. (2026). Salvia miltiorrhiza-Derived Vesicle-like Nanoparticles Functionalised Hydrogel with Excellent Ability of Oxidative Stress Modulation and Anti-Cardiomyocyte Apoptosis for Sepsis-Induced Myocardial Injury. Plant Biotechnol. J..

[63] Liu T., Wang H., Wang R., Jin Y., Wang Y., Wang S., Li X., Wang Y., Zhang Z., Su P. (2026). American ginseng-derived extracellular vesicle-like nanoparticles (AGELNs) mitigate doxorubicin-induced cardiotoxicity by inhibiting GPX4-mediated ferroptosis. Phytomedicine.

[64] Ferrucci L., Fabbri E. (2018). Inflammageing: Chronic inflammation in ageing, cardiovascular disease, and frailty. Nat. Rev. Cardiol..

[65] Luo M., Zhao F., Cheng H., Su M., Wang Y. (2024). Macrophage polarization: An important role in inflammatory diseases. Front. Immunol..

[66] Wu J., Ma X., Lu Y., Zhang T., Du Z., Xu J., You J., Chen N., Deng X., Wu J. (2022). Edible Pueraria lobata-Derived Exosomes Promote M2 Macrophage Polarization. Molecules.

[67] Liu B., Li X., Yu H., Shi X., Zhou Y., Alvarez S., Naldrett M.J., Kachman S.D., Ro S.H., Sun X. (2021). Therapeutic potential of garlic chive-derived vesicle-like nanoparticles in NLRP3 inflammasome-mediated inflammatory diseases. Theranostics.

[68] Chen X., Zhou Y., Yu J. (2019). Exosome-like Nanoparticles from Ginger Rhizomes Inhibited NLRP3 Inflammasome Activation. Mol. Pharm..

[69] Petrucci G., Rizzi A., Hatem D., Tosti G., Rocca B., Pitocco D. (2022). Role of Oxidative Stress in the Pathogenesis of Atherothrombotic Diseases. Antioxidants.

[70] Li Y., Wang X., Li S., Wang L., Ding N., She Y., Li C. (2025). Therapeutic Effects of Natural Products in the Treatment of Chronic Diseases: The Role in Regulating KEAP1-NRF2 Pathway. Am. J. Chin. Med..

[71] Li S., Ye Z., Zhao L., Yao Y., Zhou Z. (2023). Evaluation of Antioxidant Activity and Drug Delivery Potential of Cell-Derived Extracellular Vesicles from Citrus reticulata Blanco cv. ‘Dahongpao’. Antioxidants.

[72] Ijaz A., Yarlagadda B., Orecchioni M. (2025). Foamy macrophages in atherosclerosis: Unraveling the balance between pro- and anti-inflammatory roles in disease progression. Front. Cardiovasc. Med..

[73] Xie M., Li X., Chen L., Zhang Y., Chen L., Hua H., Qi J. (2025). The crosstalks between vascular endothelial cells, vascular smooth muscle cells, and adventitial fibroblasts in vascular remodeling. Life Sci..

[74] Tang H.Y., Chen A.Q., Zhang H., Gao X.F., Kong X.Q., Zhang J.J. (2022). Vascular Smooth Muscle Cells Phenotypic Switching in Cardiovascular Diseases. Cells.

[75] Teng Y., He J., Zhong Q., Zhang Y., Lu Z., Guan T., Pan Y., Luo X., Feng W., Ou C. (2022). Grape exosome-like nanoparticles: A potential therapeutic strategy for vascular calcification. Front. Pharmacol..

[76] Bennett M.R., Sinha S., Owens G.K. (2016). Vascular Smooth Muscle Cells in Atherosclerosis. Circ. Res..

[77] Wang X., He B. (2024). Endothelial dysfunction: Molecular mechanisms and clinical implications. MedComm.

[78] Wu W., Li X., Zuo G., Pu J., Wu X., Chen S. (2018). The Role of Angiogenesis in Coronary Artery Disease: A Double-Edged Sword: Intraplaque Angiogenesis in Physiopathology and Therapeutic Angiogenesis for Treatment. Curr. Pharm. Des..

[79] Sellke F.W., Li J., Stamler A., Lopez J.J., Thomas K.A., Simons M. (1996). Angiogenesis induced by acidic fibroblast growth factor as an alternative method of revascularization for chronic myocardial ischemia. Surgery.

[80] Del Re D.P., Amgalan D., Linkermann A., Liu Q., Kitsis R.N. (2019). Fundamental Mechanisms of Regulated Cell Death and Implications for Heart Disease. Physiol. Rev..

[81] Cong L., Bai Y., Guo Z. (2022). The crosstalk among autophagy, apoptosis, and pyroptosis in cardiovascular disease. Front. Cardiovasc. Med..

[82] Lian M.Q., Chng W.H., Liang J., Yeo H.Q., Lee C.K., Belaid M., Tollemeto M., Wacker M.G., Czarny B., Pastorin G. (2022). Plant-derived extracellular vesicles: Recent advancements and current challenges on their use for biomedical applications. J. Extracell. Vesicles.

[83] Rajasekaran B., Lo K.J., Pan M.H. (2026). Recent advances in PDEVs as nanocarriers for drug delivery: Loading techniques, engineering strategies and future directions. Expert Opin. Drug Deliv..

[84] Kürtösi B., Kazsoki A., Zelkó R. (2024). A Systematic Review on Plant-Derived Extracellular Vesicles as Drug Delivery Systems. Int. J. Mol. Sci..

[85] Hu Y., Zhang W., Ali S.R., Takeda K., Vahl T.P., Zhu D., Hong Y., Cheng K. (2025). Extracellular vesicle therapeutics for cardiac repair. J. Mol. Cell. Cardiol..

[86] Wang X., Liu Y., Li W., Hao J., Zhao Z., Fan H., Wu X., Liu X., Xu H., Yu T. (2026). Coptis chinensis extracellular vesicles loaded with CA1-siRNA promote endothelial repair and stent restenosis therapy by regulating the PADI2 and NF-κB pathway. J. Nanobiotechnol..

[87] Kang M., Jordan V., Blenkiron C., Chamley L.W. (2021). Biodistribution of extracellular vesicles following administration into animals: A systematic review. J. Extracell. Vesicles.

[88] Castro R., Adair J.H., Mastro A.M., Neuberger T., Matters G.L. (2024). VCAM-1-targeted nanoparticles to diagnose, monitor and treat atherosclerosis. Nanomedicine.

[89] Blindt R., Vogt F., Astafieva I., Fach C., Hristov M., Krott N., Seitz B., Kapurniotu A., Kwok C., Dewor M. (2006). A novel drug-eluting stent coated with an integrin-binding cyclic Arg-Gly-Asp peptide inhibits neointimal hyperplasia by recruiting endothelial progenitor cells. J. Am. Coll. Cardiol..

[90] Miyoshi M., Shimosato T., Takaya T. (2024). Myogenic Anti-Nucleolin Aptamer iSN04 Inhibits Proliferation and Promotes Differentiation of Vascular Smooth Muscle Cells. Biomolecules.

[91] Wang X., Chen Y., Zhao Z., Meng Q., Yu Y., Sun J., Yang Z., Chen Y., Li J., Ma T. (2018). Engineered Exosomes with Ischemic Myocardium-Targeting Peptide for Targeted Therapy in Myocardial Infarction. J. Am. Heart Assoc..

[92] Sullivan H.L., Gianneschi N.C., Christman K.L. (2021). Targeted nanoscale therapeutics for myocardial infarction. Biomater. Sci..

[93] Lee G.Y., Kim J.H., Choi K.Y., Yoon H.Y., Kim K., Kwon I.C., Choi K., Lee B.H., Park J.H., Kim I.S. (2015). Hyaluronic acid nanoparticles for active targeting atherosclerosis. Biomaterials.

[94] Zhang J., Nie S., Zu Y., Abbasi M., Cao J., Li C., Wu D., Labib S., Brackee G., Shen C.L. (2019). Anti-atherogenic effects of CD36-targeted epigallocatechin gallate-loaded nanoparticles. J. Control. Release.

[95] Dhanasekara C.S., Zhang J., Nie S., Li G., Fan Z., Wang S. (2021). Nanoparticles target intimal macrophages in atherosclerotic lesions. Nanomedicine.

[96] He Z., Luo Y., Yang S., Shi H., Huang Y.C., Zhou Z., Liu S., Zeng W., Liu W.C., Li Y. (2026). Reprogramming Lesional Macrophage Homeostasis via Interferon Regulatory Factor 5 Targeted siRNA Nanoimmunotherapy for Atherosclerosis. ACS Nano.

[97] Tang D., Wang Y., Wijaya A., Liu B., Maruf A., Wang J., Xu J., Liao X., Wu W., Wang G. (2021). ROS-responsive biomimetic nanoparticles for potential application in targeted anti-atherosclerosis. Regen. Biomater..

[98] Cheng N., Luo Q., Yang Y., Shao N., Nie T., Deng X., Chen J., Zhang S., Huang Y., Hu K. (2025). Injectable pH Responsive Conductive Hydrogel for Intelligent Delivery of Metformin and Exosomes to Enhance Cardiac Repair after Myocardial Ischemia-Reperfusion Injury. Adv. Sci..

[99] Li M., Zhao B., Liu J. (2026). Endogenous and exogenous stimuli-driven intelligent nanocarriers: Emerging strategies for the treatment of myocardial infarction. J. Nanobiotechnol..

[100] Senoner T., Dichtl W. (2019). Oxidative Stress in Cardiovascular Diseases: Still a Therapeutic Target. Nutrients.

[101] Silvis M., Kaffka Genaamd Dengler S.E., Odille C.A., Mishra M., van der Kaaij N.P., Doevendans P.A., Sluijter J., de Kleijn D., de Jager S., Bosch L. (2020). Damage-Associated Molecular Patterns in Myocardial Infarction and Heart Transplantation: The Road to Translational Success. Front. Immunol..

[102] Liu H., Deng Y., Li J., Lin W., Liu C., Yang X., Zhou Z., Jiang Y. (2025). Ginger-derived exosome-like nanoparticles: A representative of plant-based natural nanostructured drug delivery system. Front. Bioeng. Biotechnol..

[103] Lu X., Xu Z., Shu F., Wang Y., Han Y., Yang X., Shi P., Fan C., Wang L., Yu F. (2024). Reactive Oxygen Species Responsive Multifunctional Fusion Extracellular Nanovesicles: Prospective Treatments for Acute Heart Transplant Rejection. Adv. Mater..

[104] Öörni K., Rajamäki K., Nguyen S.D., Lähdesmäki K., Plihtari R., Lee-Rueckert M., Kovanen P.T. (2015). Acidification of the intimal fluid: The perfect storm for atherogenesis. J. Lipid Res..

[105] Luo T., Zhang Z., Xu J., Liu H., Cai L., Huang G., Wang C., Chen Y., Xia L., Ding X. (2023). Atherosclerosis treatment with nanoagent: Potential targets, stimulus signals and drug delivery mechanisms. Front. Bioeng. Biotechnol..

[106] Li X., Xu Z. (2025). Applications of Matrix Metalloproteinase-9-Related Nanomedicines in Tumors and Vascular Diseases. Pharmaceutics.

[107] Tapeinos C., Gao H., Bauleth-Ramos T., Santos H.A. (2022). Progress in Stimuli-Responsive Biomaterials for Treating Cardiovascular and Cerebrovascular Diseases. Small.

[108] He J., Zhang W., Zhou X., Xu F., Zou J., Zhang Q., Zhao Y., He H., Yang H., Liu J. (2023). Reactive oxygen species (ROS)-responsive size-reducible nanoassemblies for deeper atherosclerotic plaque penetration and enhanced macrophage-targeted drug delivery. Bioact. Mater..

[109] Lin Y., Liu J., Chong S.Y., Ting H.J., Tang X., Yang L., Zhang S., Qi X., Pei P., Yi Z. (2024). Dual-Function Nanoscale Coordination Polymer Nanoparticles for Targeted Diagnosis and Therapeutic Delivery in Atherosclerosis. Small.

[110] Zhang L., He F., Gao L., Cong M., Sun J., Xu J., Wang Y., Hu Y., Asghar S., Hu L. (2021). Engineering Exosome-like Nanovesicles Derived from *Asparagus cochinchinensis* Can Inhibit the Proliferation of Hepatocellular Carcinoma Cells with Better Safety Profile. Int. J. Nanomed..

[111] He Y., Huang Z., Liang J., Ji H., Pu Z. (2026). Synergistic effects of biomaterials and extracellular vesicles in treating myocardial infarction: A systematic review of preclinical studies. Acta Biomater..

[112] Riau A.K., Ong H.S., Yam G., Mehta J.S. (2019). Sustained Delivery System for Stem Cell-Derived Exosomes. Front. Pharmacol..

[113] Mol E.A., Lei Z., Roefs M.T., Bakker M.H., Goumans M.J., Doevendans P.A., Dankers P., Vader P., Sluijter J. (2019). Injectable Su-pramolecular Ureidopyrimidinone Hydrogels Provide Sustained Release of Extracellular Vesicle Therapeutics. Adv. Healthc. Mater..

[114] Rodríguez de Lope M.M., Sánchez-Pajares I.R., Herranz E., López-Vázquez C.M., González-Moro A., Rivera-Tenorio A., González-Sanz C., Sacristán S., Chicano-Gálvez E., de la Cuesta F. (2025). A Compendium of Bona Fide Reference Markers for Genuine Plant Extracellular Vesicles and Their Degree of Phylogenetic Conservation. J. Extracell. Vesicles.

[115] Pinedo M., de la Canal L., de Marcos Lousa C. (2021). A call for Rigor and standardization in plant extracellular vesicle research. J. Extracell. Vesicles.

[116] Limongi T., Milla P., Stella B., Arpicco S. (2026). Regulatory Challenges and Opportunities for Cell-Derived Extracellular Vesicles in Pharmaceutical Development: A European and Global Perspective. J. Extracell. Vesicles.

[117] Deng H., Zhang Y.W., Zhao Q.F., Huang Z.J. (2026). Medicinal Plant-Derived Exosome-like Nanoparticles: From Basic Research to Bio-medical Applications. Pharmaceutics.

[118] Rutter B.D., Innes R.W. (2020). Growing pains: Addressing the pitfalls of plant extracellular vesicle research. New Phytol..

[119] Segneanu A.E., Mogoşanu G.D., Bejenaru C., Kostici R., Bejenaru L.E. (2026). Plant-Derived Nanocarriers for Drug Delivery: A Unified Framework Integrating Extracellular Vesicles, Engineered Phytocarriers, Hybrid Platforms, and Bioinspired Systems. Plants.

[120] Shanthi K.B., Pratiwi F.W., Naillat F., Mammadova R., Sarpola M., Wu S.H., Suokas M., Jokipii-Lukkari S., Kaisto S., Samoylenko A. (2025). Cloudberry-derived nanovesicles as stable oral drug delivery systems: Gastrointestinal stability and age-related biodistribution in mice. Nanoscale.

[121] Naselli F., Volpes S., Cardinale P.S., Palumbo F.S., Cancilla F., Lopresti F., Villanova V., Girgenti A., Nuzzo D., Caradonna F. (2024). New Nanovesicles from Prickly Pear Fruit Juice: A Resource with Antioxidant, Anti-Inflammatory, and Nutrigenomic Properties. Cells.

[122] Umezawa M., Suyama F., Tachibana K., Onoda A., Takeda K. (2025). Plant-Derived Nanovesicle Enhanced Microribonucleic Acid (Mi-croRNA) Transfer from Nasal Cavity to the Brain. Mol. Pharm..

[123] Naseem M.T., Zaman W. (2026). Toxicology and biodistribution of plant-derived extracellular vesicles for drug delivery: Quality control, safety mechanisms, and translational testing priorities. Toxicol. Lett..

[124] Tao M., Fang X., Liang Z., Zhang Z., Gong W., Tan X., Zeng P. (2026). Plant-derived extracellular vesicles as a promising therapeutic and drug delivery strategy for tumor oxidative stress and inflammation. Discov. Nano.

[125] Qiang W., Li J., Ruan R., Li Q., Zhang X., Yan A., Zhu H. (2024). Plant-derived extracellular vesicles as a promising anti-tumor approach: A comprehensive assessment of effectiveness, safety, and mechanisms. Phytomedicine.

[126] Pomatto M., Gai C., Negro F., Massari L., Deregibus M.C., Grange C., De Rosa F.G., Camussi G. (2023). Plant-Derived Extracellular Vesicles as a Delivery Platform for RNA-Based Vaccine: Feasibility Study of an Oral and Intranasal SARS-CoV-2 Vaccine. Pharmaceutics.

